# 70 years of decoloniality: epistemic disobedience and global public health

**DOI:** 10.3389/fpubh.2025.1658591

**Published:** 2025-09-09

**Authors:** Justine McGovern, Lisa Fusco

**Affiliations:** Lehman College, City University of New York, Bronx, NY, United States

**Keywords:** epistemology, decolonization, global health, theory, research, practice

## Abstract

The term “epistemic disobedience” was coined by Walter Mignolo in his 2009 article entitled Epistemic Disobedience, Independent Thought and De-Colonial Freedom. While the decolonialization of global health research and practice has gained traction, epistemic disobedience is an emergent perspective in scholarship occurring at the intersection of public health and the population movement. Linked to decoloniality, a practice that coalesced in 1955, epistemic disobedience refers to research practices and perspectives that dismantle the power dynamics of colonialism by de-linking geopolitics from knowledge building (Mignolo, 2009). This mini review defines and situates key terms in historical and current literature; critically explores the usage of epistemic disobedience in public health scholarship; draws on case examples to suggest ways to apply epistemic disobedience; and articulates applications and implications in public health research and practice that seek to increase health equity as a human right.

## Introduction

The scope of scholarship examining decoloniality and Argentinian semiotician Walter Mignolo’s contributions to the discourse is too vast to address here. This review focuses on Mignolo’s concept of epistemic disobedience, a term that began to garner attention across disciplines in 2009 with the publication of his article entitled Epistemic Disobedience, Independent Thought and De-Colonial Freedom in the journal *Theory, Culture & Society.* Writing in Spanish and English, Walter Mignolo has been a key contributor for the past 30 years to theories seeking to deconstruct, challenge, and provide alternatives to colonial knowledge-building endeavors and epistemologies [i.e., (1–3)]. His ideas have been applied across a range of disciplines, including global health, education, political philosophy, human rights, art, music, ethnic and racial studies, anthropology, social work and more, as well as across the world [i.e., (4–12)]. However, public health scholarship has yet to embrace Mignolo’s theory to the extent that other fields have done so, including global health [i.e., (11, 34)].

### Distinguishing between public health and global health

The WHO refers to public health as organized efforts promoting prevention, health, and longevity with wellbeing as a goal across the physical, mental, and social domains and a focus on populations rather than individuals and diseases (13). Public health is, in large part, a distinct area of research and practice from global health. Public health consists primarily of science and practice that seeks to promote health, reduce health disparities, and identify and assess determinants of health (14). While public health may adopt an international lens and explore health topics affecting populations in different places across the world, it is not typically as engaged with the interactions between places and populations, and their mutual impact on one another (14).

In contrast, global health addresses health problems from a global perspective (15). As defined by the WHO, global health focuses on improving health and promoting health equity for populations worldwide. As a field of study and practice that informs policy, global health is concerned with health issues that transcend borders, have an impact on political and economic outcomes, and reflect geopolitical patterns (15).

While there is overlap between the domains of public health and global health, some differences remain. Significant to this review, a Google Scholar search of the terms “decoloniality and public health” yields a selection of articles referring to global health rather than public health, highlighting the different foci of the two disciplines.

### From decoloniality to epistemic disobedience

Mignolo identifies the origins of decoloniality as taking root during the Bandung Conference of 1955 (3). Over the course of the conference, representatives from 29 countries, mostly Asian and African, gathered to define a future that was tied neither to capitalism nor communism, the systems that supported their colonization. The publication of Frantz Fanon’s The Wretched of the Earth in 1961 extended the decolonial perspective into the field of psychology, adding a new angle to the discourse. Expanding on the structural-political impact of colonialism, (16) explored the dehumanizing effects of colonialism on individuals and societies. A seminal voice in decolonization scholarship and politics, Fanon called for a social movement to free individuals and societies of the long reach of colonialism (16, 17). That same year, the Conference of Non-Aligned Countries occurred in Belgrade, cementing the goals of decolonization and the commitment to de-linking from the hegemony of Western knowledge imperialism (3).

Now, 70 years after the Bandung Conference, decoloniality has come to be understood as a critique of Western systems, structures, and institutions that privilege certain societies, languages, ways of knowing, ethnicities, and other socio-political markers over others (18). Moreover, decoloniality challenges knowledge and knowledge production that stem from colonial practices (19, 20). According to Mignolo (3), the focus of decoloniality in its current iteration is on social justice and global equity across domains, including the health, social, economic, and epistemic spheres, as well as across locations. Mignolo (3) has been instrumental in recasting decoloniality as necessary not only in identifying colonized locations (i.e., Fanon’s Martinique), but also in the population movement itself, that is, migration. He suggests that migration patterns force a variety of consciousnesses to interact, potentially causing conflict. These interactions and conflicts not only result in disparities and inequities when systems and institutions privilege one epistemology over the other, but also highlight the need to unseat practices that promote colonial dominance. By challenging both the knowledge and knowledge-building endeavors of the colonial enterprise, epistemic disobedience offers an alternative that opens doors to other epistemic outcomes (2, 3). Over approximately the past 20 years, scholarship exploring epistemic disobedience and North and South interactions and exchanges in service to decolonizing global health has flourished [i.e., (21–23)]. The entirety of the literature is too broad to address in this mini review. Several scoping reviews provide a starting point for those interested in more breadth and depth [i.e., (24, 25)].

## Applied epistemic disobedience

### Knowledge production

Consisting of the de-linking of knowledge and the production of knowledge from geo-politics, epistemic disobedience aims to unlock new epistemologies about people, places, processes, and possibilities that extend beyond the tropes imposed by colonial systems that privilege certain sources of knowledge (i.e., statistics instead of storytelling or dreams); places of knowledge (Eurocentric locations instead of the Global South); and products of knowledge (scholarly texts instead of art) (2, 11). To accomplish this, it relies on research practices that destabilize current power structures at both macro and micro levels (26, 27).

Naidu (11) describes some macro attempts at levelling the hierarchies of knowledge. She cites mentoring and training Global South authors in how to meet the standards of Global North academic journals; publishing special issues of academic journals that focus on Global South voices; and creating regional, specialized editions of journals and conferences (11). Epistemic disobedience at the macro level can include research methodologies as well. For example, participant action research (PAR) and photovoice, which not only promote the co-creation of knowledge with citizen-scientists (no longer referred to as research subjects) but also disseminate findings in non-traditional means, can be considered examples of applied epistemic disobedience [i.e., (9, 10)].

Other practices more focused on the micro-production of scholarship have also gained traction in research that seeks to challenge colonial epistemologies. The inclusion of reflexivity and positionality statements in research narratives is now commonplace in decolonized qualitative research (28). Defined as a form of critical thinking that seeks to reveal assumptions while also finding ways to dismantle these assumptions, a reflexivity statement is distinct from a positionality statement (28). Practicing reflexivity requires that the researcher reflect and comment on how his/her role might influence research processes. Moreover, it situates the researcher as a learner, not only about the topic at hand but also about the self (28). While reflexivity practice has been criticized for replicating the very tropes it aims to challenge because it runs the risk of re-centering the researcher’s voice and experience, it can also be an effective tool in exploring epistemological assumptions and privilege biases (27). Often linked to reflexivity, positionality more specifically asks researchers to examine their identity and social capital in relation to the study population (28). These statements constitute a manifestation of epistemic disobedience because they counter the notion of the researcher as the expert; reject that knowledge-building is an objective pursuit free of prejudice; and refute that epistemologies are immutable (26, 29).

### Knowledge dissemination

Berdai Chouani et al. (29) stress that seeking alternative ways to disseminate knowledge is essential because systems of publication themselves reflect colonial priorities. For example, scientific journals continue to privilege quantitative research, which prizes objectivity, statistical methods, and peer review, and English has become the lingua franca of scholarly articles. These standards reinforce colonial tropes by ignoring inherent hierarchies of research that tee-up scholars with access to top-level education, preparation, and networks for success in academia (2, 29). Finding alternative ways to disseminate findings, including the language in which findings are shared, constitutes an additional form of epistemic disobedience (2, 11). Publishing widely in Spanish and English himself, Mignolo (2) and other scholars advocating for epistemic disobedience argue for broadening the languages of research as well as the range of what should be considered data, of products that can communicate findings, and of methodologies.

## Epistemic disobedience across disciplines

While references to epistemic disobedience in public health are virtually absent, many are readily available in other fields. The range of examples is too broad to cover here. Instead, examples from two fields, education and the arts, are included.

In education, Ramirez (30) applies epistemic disobedience in at least two ways. She includes a positionality statement and writes in the first person. Together, these narrative choices challenge the colonial notion of objectivity by asserting the authority of the researcher. In another example of epistemic disobedience from the field of education, Carr et al. (31) use participatory methods based on engaged listening, and the article contains positionality statements from each of the seven co-authors. Moreover, the article includes an abstract in Spanish, despite being published in English.

Bridging the fields of education and art, Hadeer’s (8) article on slow photography offers an example of epistemic disobedience in data collection and knowledge dissemination. In her own words:

My Slow Photography practice requires me to be fully attentive to everyday life, which gives voice to the present, the ordinary, and the unknown. Slow Photography is situated in holistic ways of thinking. By delinking my future- oriented, top-down, and separateness epistemology, I am cultivating my capability to do educational research that can reflect teachers and students’ struggle. I aim to find hope behind the numbers, labels, and silence that are caused by the suppression of colonial bureaucratic institutions. (p. 75)

In another example from the arts, Miles (32) expounded on epistemic disobedience in contemporary Latin American art. While this study is traditional in its presentation, it extends the reach of epistemic disobedience by applying it to artists challenging colonial tropes in their practice. Their art becomes both a methodology to accomplish epistemic disobedience and an outcome of its application.

## Discussion

Coined in 2009 by Walter Mignolo, epistemic disobedience refers to the de-linking of knowledge production and knowledge itself from geopolitics. It claims that colonial practices have ignored the relationships between power, politics, and privilege in the development of epistemologies. As a result, epistemologies currently promote ethnicity-based disparities that need to be addressed and redressed (2). This mini review explores the relevance and manifestations of epistemic disobedience in public health.

Unfortunately, unlike its cousin, global health, public health has not applied processes of decoloniality to a large extent, and epistemic disobedience has not emerged as a common topic in either theoretical or research articles in public health. This is not to say that public health researchers are not embedding epistemic disobedience methods in their study. Reflexivity and positionality statements, participatory action research methods, and structural changes, such as publishing special issues of journals centering on new voices, are now commonplace in public health and other fields. However, global health, education, the arts, political philosophy, anthropology, and other disciplines have taken the lead. Not only do these disciplines provide examples of the framework in action, but they also contribute critical theoretical perspectives to decolonial knowledge-building endeavors.

### Next steps

Focusing on global health, Naidu (33) suggests some strategies for embedding epistemic disobedience in scholarship and decolonizing the field. Specifically, she highlights the blind eye of institutions and systems that privilege academic, political, economic, and Eurocentric perspectives on global health, rather than social, personal, emotional, and survival-based indigenous perspectives on health care workers, a majority of whom are women of color, regardless of where they practice (33). Naidu (33) has articulated guidelines to move forward with the decolonization project. These provide the next steps. In her own words:

Requirements for resistance in global health scholarship.

• Editorial boards declaring their gender, racial, colonial, and ancestral privileges and experiences in low-income and middle-income countries (LMICs).

• Authors claiming space to write with emotion about personal and local identity perspectives in global health.

• Supporting editors from diverse spaces with the administration of editorial work, while trusting, protecting and advancing the value of their previously neglected racial, gender, historical, and personal perspectives in editorial decision making.

• Promoting abstracts in LMIC authors’ first languages and the Indigenous languages of locations where research is done.

• Establishing multiple platforms and methods of constructing and presenting research and findings within global health scholarship.

• Relieving LMIC scholars of the pressure to provide solutions to problems they did not create, offering instead an opportunity for dialogue on equal terms. (p. e1334)

While Naidu (33) articulates structural strategies for decolonizing knowledge building and dissemination in the context of global health, other steps are required as well. Developing more examples of epistemic disobedience and raising awareness about the need to decolonize public health scholarship are two such steps. Future explorations into how and how often epistemic disobedience is made manifest in research endeavors—and who is engaged in the practice—are necessary to fill knowledge gaps about the framework. Taking these steps might inspire more scholars to adopt the framework and contribute to reducing the impact of colonial power differentials that plague epistemological knowledge building in public health. Ultimately, Naidu’s (33) call for resistance stands to increase health equity and decrease the legacy of racism in public health research in the global context.
